# Cytokine changes in cerebrospinal fluid and plasma after emergency orthopaedic surgery

**DOI:** 10.1038/s41598-022-06034-9

**Published:** 2022-02-09

**Authors:** Michael Fertleman, Christopher Pereira, Melanie Dani, Benjamin H. L. Harris, Matteo Di Giovannantonio, Simon D. Taylor-Robinson

**Affiliations:** 1 Cutrale Perioperative and Ageing Group, Department of Bioengineering, Imperial College London, London, UK; 2 Computational Biology and Integrative Genomics, Department of Oncology, University of Oxford, Oxford, UK; 3 Department of Surgery and Cancer, Imperial College London, London, UK

**Keywords:** Cytokines, Blood-brain barrier

## Abstract

Neuroinflammation after surgery and its contribution to peri-operative neurocognitive disorders (PND) is not well understood. Studying the association between central and peripheral cytokines and neuroinflammation is a prelude to the development of treatments for PND. Here, we investigate the hypotheses that there is a greater cytokine response in cerebrospinal fluid (CSF) than plasma after orthopaedic surgery, and that plasma cytokine levels are directly related to CSF cytokine levels, indicating that plasma cytokine levels may have potential as biomarkers of neuroinflammation. Patients admitted with a fractured neck of femur were invited to participate in this study. Participants had a spinal catheter inserted just prior to induction of anaesthesia. Samples of blood and CSF were taken before, immediately after, and on the first day following emergency surgery. The catheter was then removed. Samples were analysed for the presence of ten cytokines by immunoassay. A spinal catheter was successfully inserted in 11 participants during the 18-month study period. Five plasma cytokines (IL-4, IL-6, IL-10, IL-12p70 and IL-13) rose significantly following surgery, whereas all ten CSF cytokines rose significantly (IL-1β, IL-2, IL-4, IL-6, IL-8, IL-10, IL-12p70, IL-13, IFN-γ and TNF-α) (adjusted-*p* < 0.05). Central (CSF) cytokine levels were consistently higher than their peripheral (plasma) counterparts after surgery, with some patients having a particularly marked neuroinflammatory response. The greatest increases occurred in IL-8 in CSF and IL-6 in plasma. There were significant, strong positive correlations between several of the measured cytokines in the CSF after surgery, but far fewer in plasma. There was no significant correlation between cytokine levels in the plasma and CSF at each of the three time points. To our knowledge, this is the first study to analyse paired samples of plasma and CSF for cytokine levels before and after emergency orthopaedic surgery. This study demonstrates that following surgery for a fractured neck of femur, there is a far greater rise in cytokines in the CSF compared to plasma. The lack of correlation between peripheral and central cytokines suggests measurement of peripheral cytokines are not necessarily related to which patients may have a large neuroinflammatory response.

## Introduction

### Neuroinflammation

There is a growing appreciation that the immune system affects the central nervous system (CNS) after surgical trauma, which may lead to peri-operative neurocognitive disorders (PND), including delirium^1^. However, the mechanisms involved are unclear.

One hypothesis to explain PND is that patients undergoing surgery develop an accentuated inflammatory response peripherally, causing the release of pro-inflammatory cytokines from macrophages. These cytokines may then cross the blood–brain barrier (BBB), activating microglial cells^2^. Microglia are the predominant immune cell in the brain, making up around 5–10% of cells in the CNS^3^. They are responsible for immune surveillance and monitoring, and become activated in response to insult or injury^1^. Once activated, similar inflammatory responses are seen to macrophages^4^. This inflammatory response includes the release of cytokines, which can have direct toxic effects on the surrounding cells and drive the inflammatory response of astrocytes, which may result in weakening of the BBB^5^, thus making the CNS even more vulnerable to peripheral cytokine signals. The BBB normally prevents factors in the systemic circulation from disrupting normal neuronal activity^6^. However, disruption to the BBB due to neurodegenerative disorders or ageing can weaken this barrier, potentially leaving the brain vulnerable to chemical changes in the systemic circulation^7^.

Cytokines are a group of inflammatory mediators that play a key role in the process of neuroinflammation^8^. Some cytokines tend to have a pro-inflammatory role; IL-1β, IL-6 and IL-8, while others, such as IL-4, IL-10 and IL-13, tend to be anti-inflammatory^9^. Studies on elective surgical patients have shown that there are greater post-operative cytokine increases in the cerebral spinal fluid (CSF) than in plasma^10,11^. There is also some evidence to suggest that CSF cytokine changes may be associated with PND^12^. A study of ten patients undergoing elective major knee surgery found that a greater rise in the CSF cytokines IL-6 and IL-8 occurred in the one patient who developed delirium^10^. Furthermore, in a study of 24 patients undergoing either elective hip or knee surgery, those patients with a poor neurocognitive outcome had higher post-operative CSF levels of IL-6 and IL-8^13^. While these studies are small, they suggest a potential role for neuroinflammation in worse cognitive outcomes following surgery.

Noting above, little is known about PND and its relationship to cytokines in the emergency setting. Fractured neck of femur is the most common serious injury in older people, and the commonest cause of death following an accident, with a mortality rate of 6.1% in the first month^14^. The commonest complication of any surgery in older people is delirium, which affects a quarter of patients with a fractured neck of femur^14^. The strongest risk factors for developing delirium include advancing age and pre-existing cognitive impairment^15^. Delirium in patients with a fractured neck of femur and dementia also significantly increases 1-year mortality^16^.

Previous studies in patients with a fractured neck of femur have only examined *pre-*operative CSF and plasma samples^17,18,19^. To our knowledge, no study has looked at cytokine changes in patients undergoing emergency orthopaedic surgery for a fractured neck of femur who had both CSF and plasma samples collected in the pre- and post-operative period.

The aim of this study was to evaluate the CSF and plasma inflammatory response to surgery for a fractured neck of femur in order to:

1. Determine changes in CSF and plasma cytokines before and after emergency orthopaedic surgery.
2. Investigate potential inter-correlation between ten cytokines at different time points in CSF and plasma.
3. Establish if plasma cytokines levels are directly related to the levels of CSF cytokines, thereby enabling plasma cytokines to be used as a much more convenient marker of neuroinflammation.

## Methods

This was a prospective observational study looking at peri-operative changes in cytokine levels in the CSF and plasma of patients undergoing emergency orthopaedic surgery for a fractured neck of femur.

### Study population

Patients having an emergency admission with a fractured neck of femur admitted to St Mary’s Hospital, London, UK, were considered for recruitment into the study. Recruitment took place over an 18-month period.

Exclusion criteria were:

- Recent use of antiplatelet or anticoagulant medication.
- Need for anticoagulation in the immediate post-operative setting.
- Previous lumbar spine surgery.
- Previous history of post-dural puncture headache.
- Body mass index > 30 kg/m^2^.
- If participants required the presence of a translator for recruitment into the study.
- History of alcohol or drug abuse.
- Inability to consent to surgery.
- Pre-existing cognitive impairment or Parkinson’s Disease.
- Psychotropic or corticosteroid medication use.

This study received approval from the hospital research governance team at St Mary’s Hospital, London, UK (15SM3116), and the London Stanmore Research Ethics Committee (16/LO/0183). All patients gave written, informed consent before taking part in the study, which was taken by the same author (MF). The study conformed to the precepts set out in the Declaration of Helsinki of 1975.

### Specimen collection and storage

Study participants had a spinal catheter inserted immediately before surgery by an anaesthetist. Prior to spinal catheter insertion, a subcutaneous local anaesthetic of 1% lidocaine was given at the planned site of insertion for patient comfort. Once inserted, 5 mL of CSF was withdrawn into a polypropylene tube. A blood sample was taken in an ethylenediaminetetraacetic acid (EDTA) tube at the same time point. These samples were classified in the statistical analysis as time-point one (T1). Following surgery, CSF and blood samples were collected in the operating theatre recovery area between one and four hours after surgery. An initial 2 mL of CSF, corresponding to the “dead space” in the spinal catheter was discarded before sample collection. The samples taken at this point were classified as time-point two (T2). A final set of samples was taken the morning after surgery. These samples were classified as time-point three. The spinal catheter was removed by 10am on day one post-surgery.

Once taken, samples were moved to the laboratory within 20 min of collection and centrifuged at 3000*g* for 10 min at room temperature. Each of the plasma and CSF samples were divided into two 500 μL and a further 200 μL aliquots. Samples were stored at − 80 °C until analysis.

### Patient monitoring

No formal cognitive testing was undertaken, but delirium was identified through retrospective analysis of medical records. Records were kept of routine blood tests, the anaesthetic used and the length of hospital stay. Participants were monitored contemporaneously by the same author (MF) for any side effects relating to the spinal catheter, including bleeding, infection, paralysis and a low-pressure CSF headache.

### Immunoassays

Cytokines were analysed by electrochemiluminescence using the V-PLEX Proinflammatory Panel 1 Human Kit as per the manufacturer’s instructions (Meso Scale Discovery, Maryland, USA)^20^. This kit assays IL-1β, IL-2, IL-4, IL-6, IL-8, IL-10, IL-12p70, IL-13, IFN-γ and TNF-α. The range for the lowest level of detection (LLD) was between 0.00676 pg/mL for IL-4 and 0.336 pg/mL for IL-13. If samples were above the upper limit of detection (ULD) for the assay, they were diluted and re-analysed, as per the manufacturer’s instructions. This was necessary for seven IL-8 results which were diluted at a dilution of 30:1 using Diluent 2, which was supplied with the kit assay. For cytokine levels below the LLD or detected but below the fit curve, the LLD value for the cytokine assay was used, as per a previously described method^21^. All cytokine analysis was completed in the Infectious Diseases Laboratory at Imperial College London.

### Statistical analysis

Analyses were carried out using Python software, version 3.7 (available from www.python.org). After establishing a non-gaussian distribution of data, a Friedman test with Bonferroni correction was used to look for statistically significant changes in CSF and plasma cytokines across the three time points. However, while a result may be non-significant across all three time-points, there may still be significant differences between two of the three time points. Thus, a two-sided test determined if the changes occurring between pairs of time points were significant. In these instances, a Wilcoxon Signed Rank test was used with Bonferroni correction for further stringency. Spearman’s rank correlation coefficient, again with a Bonferroni correction, assessed cytokine inter-correlation. An adjusted-*p* value of < 0.05 was considered statistically significant. A power calculation to determine the necessary sample size was not undertaken before the investigation, as the number of participants who would be able to consent and complete the study was anticipated to be small.

### Ethics approval and consent to participate

This study received approval from the hospital research governance team at St Mary’s Hospital, London, UK (15SM3116), and the London Stanmore Research Ethics Committee (16/LO/0183). All patients gave written informed consent before surgery.

## Results

During an 18-month period, 21 participants were recruited into the study. One person withdrew consent prior to spinal catheter insertion and one was excluded after being found to have suffered a subarachnoid haemorrhage. The subarachnoid haemorrhage was diagnosed on CT head scan and most likely occurred before the person was admitted to hospital, as a result of the fall that also led to their fractured neck of femur. In eight people, spinal catheter insertion was not possible due to technical difficulties, thus giving a cohort of 11 participants who completed the study.

Table 1 shows the biographical and pre-operative information for the 11 participants. Nine of the 11 participants were female, and the mean age of the participants was 78 years (SD 6 years). Three participants also had wrist fractures likely sustained at the same time as their fractured neck of femurs.

**Table 1:** Participant’s background and treatment prior to surgery.

ID	Sex	Age	Side	Pre-operative			Past Medical History	Other injury
				FIB	Opiate (mg)	AMTS		
P02	F	75	L	Y	46	7	Epilepsy	
P03	F	83	L	Y	0	7	HTN, T2DM	Wrist #
P09	F	71	R	N	20	7	HTN, IHD, Cirrhosis	Wrist #
P010	F	78	L	Y	26	10		
P011	F	82	L	Y	16	9	Aspirin use, HTN	Wrist #
P012	F	74	L	Y	10	8		
P013	F	72	L	N	18	10	HTN	
P014	M	75	L	Y	6	10		
P015	M	89	R	Y	5	10	HTN	
P019	F	76	L	Y	9	10		
P020	F	86	R	Y	9	NR	Breast cancer	

A table showing the medical backgrounds and pre-operative management for the 11 patients included in this study.

AMTS = Abbreviated Mental Test Score, IHD = Ischaemic heart disease, FIB = Fascia Iliaca block, HTN = Hypertension, NR = Not recorded, T2DM = Type 2 Diabetes, L = Left, R = Right, # = Fracture.

### Timetable of sample collection

All participants had pre-operative and post-operative day one plasma and CSF samples taken. One participant had only these two samples collected (P012). The remaining ten participants also had samples taken immediately post-surgery.

Supplementary Table S1 summarises the type of orthopaedic procedure carried out and the anaesthetic given. The mean time between admission and surgery was 30 h (SD 22 h). Where recorded, all patients had a spinal block, using the catheter which had been sited as part of the study protocol. In addition, three patients also received a general anaesthetic, with the remainder also receiving sedation.

The post-operative progress of the participants is summarised in Supplementary Table S2. The mean length of inpatient stay was 15 days (SD 11 days). There was one complication from the use of a spinal catheter, namely a low-pressure headache in participant P012, which resolved at the end of the first post-operative day.

### Cytokine changes following surgery

Table 2 shows that at all three time-points, significant rises were seen in the plasma cytokines IL-4, IL-6, IL-10 and IL-12p70 and in the CSF cytokines IL-1β, IL-2, IL-6, IL-8, IL-10, IL-13 and TNF-α.

**Table 2:** Summary of cytokine measurements in plasma and CSF.

Cytokine	T	CSF Median [IQR] (pg/mL)	CSF adjusted-*p* value Friedman test	Plasma Median [IQR] (pg/mL)	Plasma adjusted-*p* value Friedman test
IL-1β	T1 T2 T3	0.07 [0.07–0.07] 0.17 [0.11–0.31] 0.36 [0.28–1.72]	< 0.01	0.07 [0.07–0.10] 0.07 [0.07–0.09] 0.07 [0.07–0.11]	1
IL-2	T1 T2 T3	0.03 [0.03–0.05] 0.06 [0.06–0.10] 0.28 [0.07–1.15]	< 0.05	0.08 [0.04–0.12] 0.09 [0.04–0.15] 0.10 [0.06–0.17]	0.76
IL-4	T1 T2 T3	0.01 [0.01–0.01] 0.01 [0.01–0.03] 0.06 [0.02–0.11]	0.74	0.01 [0.01–0.01] 0.02 [0.01–0.02] 0.04 [0.03–0.05]	< 0.01
IL-6	T1 T2 T3	0.51 [0.40–0.80] 1.84 [1.08–10.51] 35.80 [13.03–51.98]	< 0.01	4.30 [4.00–5.95] 8.09 [6.13–9.53] 36.18 [26.33–63.90]	< 0.05
IL-8	T1 T2 T3	49.43 [34.46–61.21] 121.86 [99.48–163.90] 533.34 [283.10–4207.50]	< 0.01	6.51 [4.22–10.20] 5.28 [4.05–8.69] 7.10 [4.05–15.78]	1
IL-10	T1 T2 T3	0.08 [0.05–0.12] 0.71 [0.46–1.39] 0.92 [0.52–2.07]	< 0.01	0.43 [0.37–0.84] 1.08 [0.94–1.94] 1.15 [0.97–1.28]	< 0.05
IL-12p70	T1 T2 T3	0.03 [0.03–0.03] 0.03 [0.03–0.07] 0.22 [0.08–0.45]	0.18	0.06 [0.04–0.08] 0.06 [0.04–0.10] 0.13 [0.11–0.17]	< 0.05
IL-13	T1 T2 T3	0.43 [0.34–0.62] 0.79 [0.40–1.28] 3.47 [1.85–6.95]	< 0.05	0.34 [0.34–0.34] 0.37 [0.34–0.40] 0.43 [0.36–0.78]	0.11
IFN-γ	T1 T2 T3	0.22 [0.22–0.22] 0.27 [0.22–0.54] 0.57 [0.23–2.26]	0.85	1.92 [1.33- 2.27] 1.58 [1.07–1.93] 1.37 [0.75–1.65]	1
TNF-α	T1 T2 T3	0.12 [0.11–0.18] 0.94 [0.36–2.41] 1.53 [0.54–3.32]	< 0.01	2.83 [1.87–3.66] 2.04 [1.50–2.75] 2.86 [2.32–3.18]	1

Cytokine levels in plasma and CSF before surgery (T1), immediately after surgery (T2) and the day after surgery (T3).

The levels of plasma and CSF cytokines tended to be lower before than after surgery. In the pre-operative CSF samples, cytokines IFN-γ and IL-12p70 were mostly below the LLD, while in plasma, IL-1β and IL-13 were typically below the LLD.

**Figure 1:**
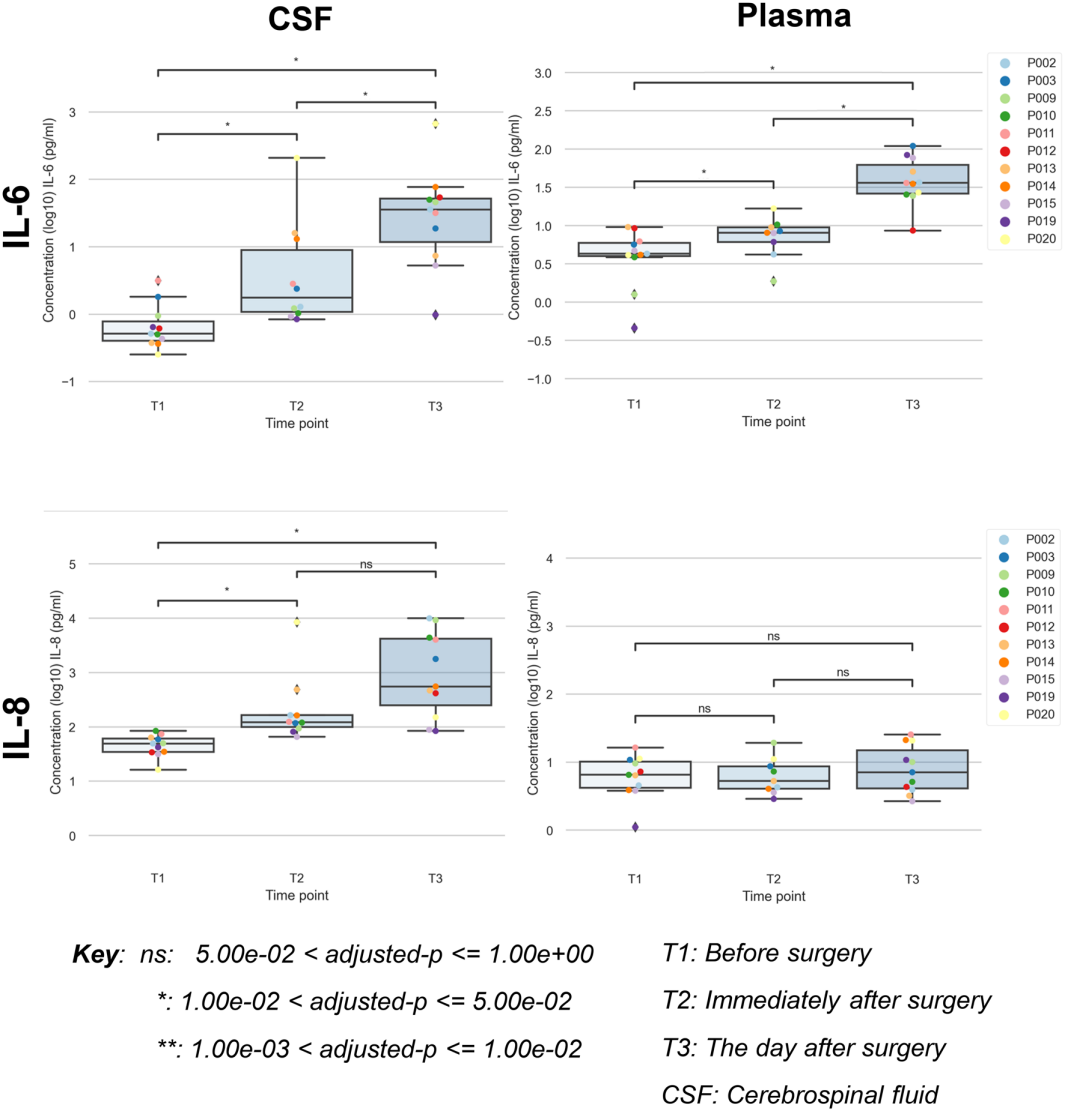
Peri-operative changes in IL-6 and IL-8 in plasma and CSF. Changes in IL-6 and IL-8 in plasma and cerebrospinal fluid (CSF) before surgery (T1), immediately after surgery (T2) and the day after surgery (T3).

Figure 1 shows box-plots for the cytokines IL-6 and IL-8 measured in the CSF and plasma of the 11 participants. The plots are for three time-points, T1 (before surgery), T2 (immediately after surgery) and T3 (morning after surgery). Each participant is represented by a colour that is consistent across the box-plots.

### Plasma cytokine findings

Between T1 and T3 there were significant increases in plasma levels of IL-4, IL-6, IL-10, IL-12p70 and IL-13, but not IL-1β, IL-2, IL-8, IFN-γ or TNFα. The most statistically significant change occurred in IL-6 between T1 and T3 (adjusted-*p* < 0.05), as shown in Fig. 1.

### CSF cytokine findings

CSF cytokines rose significantly immediately after surgery (T2) for all cytokines except for IL-4, IL-12p70, IL-13 and IFN-γ. Of these four cytokines, all but IFN-γ showed significant rises by the day after surgery (T3) when compared to baseline. The largest increase occurred in IL-8 between T1 and T3 (adjusted-*p* < 0.05). There were also large rises in IL-6 between T1 and T3 (adjusted-*p* < 0.05) with participants P014 and P020 having a particularly marked rise. Some participants’ IL-6 levels continued to rise on the day following surgery (e.g., P020), although a decline in IL-6 levels was seen in others (e.g., P013 and P014).

Following surgery, IL-2 in CSF rose from very low baseline levels, with large rises particularly in participants P014, P09 and P020. There was a rise in (anti-inflammatory) IL-10 following surgery alongside the rise in pro-inflammatory cytokines.

### Intercorrelation between cytokines

**Figure 2:**
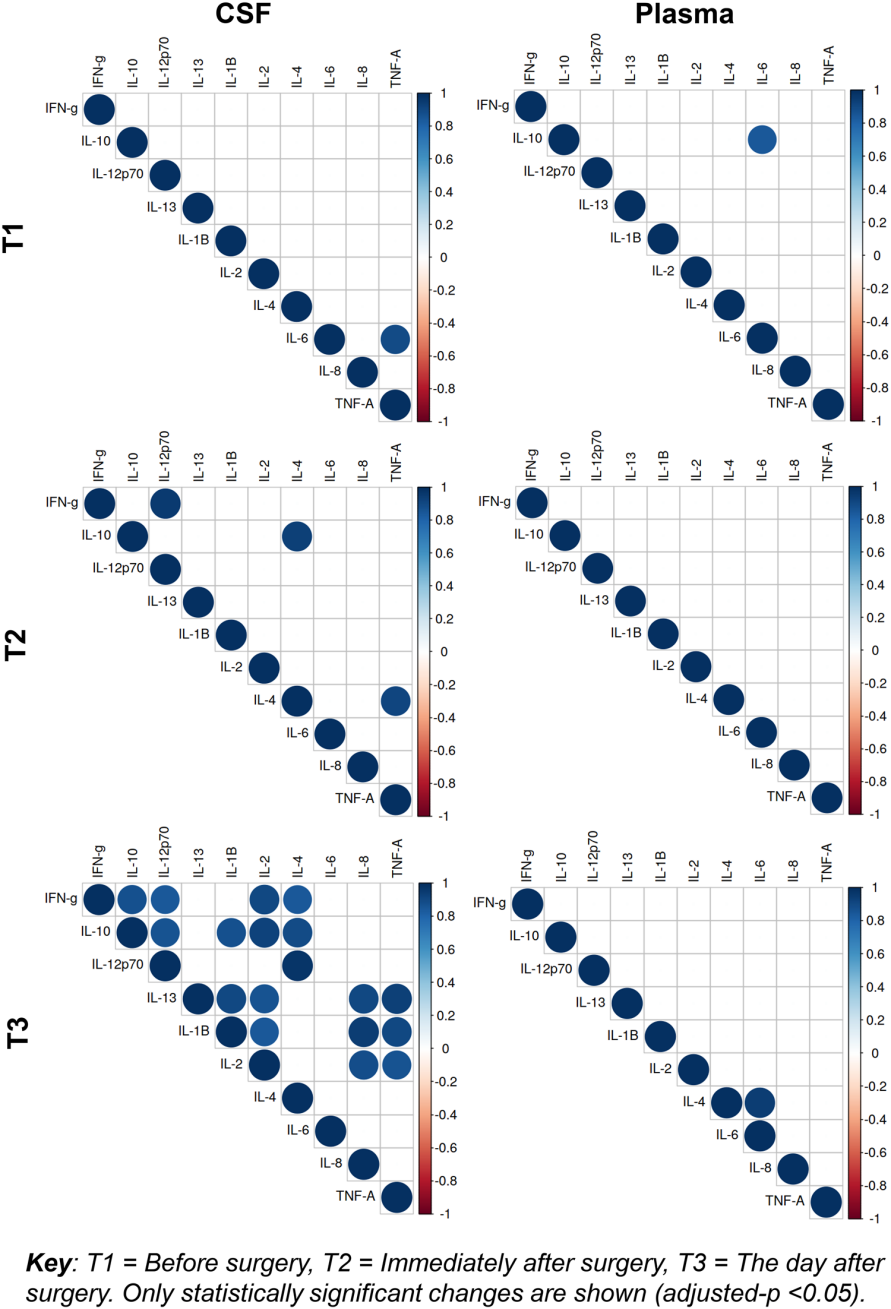
Correlation plots between cytokines at different time points. Correlation plots between cytokines for plasma and cerebrospinal fluid (CSF) before surgery (T1), immediately after surgery (T2) and the day after surgery (T3).

The correlations between the individual cytokines in the CSF and the plasma were different at each of the three time points and were more commonly found in the CSF (Fig. 2). At T1, the significant correlations observed in the CSF were between IL-6 and TNF-α (r = 0.89, adjusted-*p* < 0.001), whereas in plasma, IL-10 levels were strongly positively correlated with levels of IL-6 (r = 0.85, adjusted-*p* < 0.05). At T2, there were strong positive correlations between three pairs of cytokines in the CSF: IL-10 and IL-4 (r = 0.94, adjusted-*p* < 0.01), IL-4 and TNF-α (r = 0.91, adjusted-*p* < 0.05) and IFN-γ and IL-12p70 (r = 0.96, adjusted-*p* < 0.001). However, in plasma no significant correlations between cytokines were seen at T2. At T3, there were a number of strong positive correlations between 18 pairs of different cytokines, as shown in Supplementary Table S3. By contrast, in plasma there were significant correlations between only IL-4 and IL-6 (r = 0.95, adjusted-*p* < 0.001). Additionally, there were no significant correlations between the CSF and plasma cytokine levels at the different time points (data not shown).

## Discussion

To our knowledge, this is the first study to analyse paired samples of plasma and CSF for cytokine levels before and after emergency orthopaedic surgery. Levels of plasma and CSF cytokines were far lower prior to surgery, suggesting that the major inflammatory response does not occur as a result of fracture or pre-operative management. Consequential problems associated with fracture, such as pain, dehydration, medication use and catabolic state are, thus, not as pro-inflammatory as the effects of surgery. Surgery itself causes a rise in cytokine levels, which rise disproportionately in CSF as compared to plasma.

### Plasma cytokine findings

Of the ten measured plasma cytokines, five (IL-4, IL-6, IL-10, IL-12p70 and IL-13) increase significantly following surgery. The greatest increase was in IL-6 which rises significantly in plasma immediately following surgery and continues to rise on the day after surgery. Unlike the other cytokines measured, the level reached in the plasma was almost as high as the level of IL-6 detected in the CSF. It is probable that IL-6 plays a key role in the development of the inflammatory cascade in the genesis of delirium. However, though there was a negative correlation between plasma and CSF IL-6 at T3 (r = − 0.79), this result did not achieve significance when subjected to a correction for false discovery. It is noteworthy that, while increases in plasma were shown in IL-6, they were not shown in IL-1β, IFN-γ and TNF-α. This is of note as this group of four cytokines encompasses the most common targets of therapy in the treatment of various auto-immune diseases and are commonly raised in a variety of other inflammatory conditions^22,23^.

Plasma levels of IL-1β, IL-6, IL-8, IL-10 and TNF-α are frequently measured in other studies on patients with fractured neck of femur undergoing emergency surgery. These studies have consistently shown increases in post-operative IL-6^24,25^, as confirmed in the current study. One study of 180 patients showed an increase in post-operative IL-1β and TNF-α, which was not replicated here^26^. While the LLD for IL-1β is not available for this particular study^26^, it may be that the limited ability of the assay to detect IL-1β meant it was not possible to replicate these results. This study had a LLD of 0.0715 pg/mL for IL-1β, which is comparable to the LLD used in other studies, where it is 0.05 pg/mL^17^ and 1 pg/mL^19^. The low participant numbers in this study may have meant it was not possible to replicate the increases in post-operative IL-1β and TNF-α.

### CSF cytokine findings

Except for IL-6 and IL-8, CSF cytokines IL-1β, IL-4, IL-6, IL-10, IL-12p70, IFN-γ and TNF-α were undetectable or detected at very low levels prior to surgery. Similar to another study on fractured neck of femur patients, CSF IL-8 levels were higher in terms of concentration than other measured cytokines prior to surgery^27^.

Our study shows marked increases in IL-8 after surgery. Previous studies in patients undergoing surgery for a fractured neck of femur have analysed cytokines in CSF only before surgery^12,17,18,19^. However, studies in patients undergoing elective orthopaedic procedures and aortic valve replacement have shown similar rises in IL-8 after an operation^27,11,28^. The current study shows that CSF cytokines rise out of proportion to any elevations in the plasma. This disproportionate rise in CSF cytokines needs to be further examined and raises the question of whether CSF cytokines can be targeted peri-operatively to reduce PND. The one exception was plasma IL-6 which rose to a similar level to CSF IL-6.

### High cytokine responders

In line with other studies, we identified a subgroup of patients who develop disproportionately high CSF cytokine responses, so-called ‘high cytokine responders’^11^. Here, participants P02, P09 and P011 had the greatest CSF cytokine responses.

Patients’ clinical records were retrospectively reviewed for evidence of delirium and showed that one participant (P020) had a diagnosis of delirium after fractured neck of femur surgery. If PND indeed arises from a neuroinflammatory effect of cytokines, it is intriguing that this patient did not have the greatest CSF cytokine responses within the cohort. Future studies will be needed to prove the link between high cytokine responders and PND.

### Intercorrelation findings

Levels of cytokines in the CSF, while not exhibiting an association with each other before surgery, strongly correlate on day one after surgery at T3. In contrast, patients with high levels of one plasma cytokine did not have parallel increases in other plasma cytokines, either before or after surgery. This indicates the value of studying cytokine levels in CSF, rather than cytokine levels in blood.

As with other studies, there was no significant correlation detected between plasma and CSF cytokines^11^. This would seem to go against the theory that plasma cytokines are responsible for directly crossing the BBB, activating microglia and consequent further cytokine release^1,2^. This is despite evidence that the BBB becomes weakened in some patients with a fractured neck of femur peri-operatively^29^. However, a number of factors are known to activate microglia after surgery^1^. Animal models have shown that an injection of lipopolysaccharide results in microglia activation irrespective of systemic cytokines^30^. Furthermore, soluble triggering receptor expressed on myeloid cells 2 (sTREM2), a highly specific microglial receptor, has been shown to be increased in the CSF of patients with delirium after a fractured neck of femur^31^. This suggests that microglial activation may occur secondary to more specific ligands than just cytokines^1^. It may be that proinflammatory triggers other than the plasma cytokines measured in this study are responsible for activating microglia and driving neuroinflammation peri-operatively.

The lack of correlation between peripheral and central cytokines is disappointing, as the measurement of plasma cytokines is much simpler than the measurement of CSF cytokines, and would have been simpler to exploit clinically. In particular, the number of patients in this cohort for whom either spinal catheter insertion was contraindicated or technically not possible demonstrates the practical difficulties in obtaining CSF. It remains to be a seen whether in a healthy young population, with an intact BBB creating an immune-privileged CNS environment, there is a correlation between CSF and plasma cytokine levels.

### Limitations

This study was limited by only having eleven participants. Difficulties arose with spinal catheter insertion in eight other participants, meaning samples were not collected. Patients with a fractured neck of femur are typically older with coexisting spinal disease, making spinal catheter insertion technically difficult. Many patients were excluded from the study for not having the capacity to consent to surgery or being on antiplatelet or anticoagulant medications. The limited participant numbers likely reduced the study’s ability to identify significant cytokine patterns. Furthermore, the exclusion of patients who required a translator to give informed consent limited the diversity of the study.

Plasma and CSF samples were taken only at 2–3 time-points. This limitation was largely due to the availability of laboratory staff. Permission was not granted for use of the laboratory out of hours, limiting patient recruitment and the number of samples that could be taken. Hence it was not possible to determine detailed kinetic changes in cytokines in the peri-operative period. Future studies would need to take more frequent sampling of biofluids, while ensuring that the potential side effects of large volume CSF removal are avoided.

In this study, it was impossible to determine whether the cytokine changes occurred because of a general anaesthetic or the trauma of surgery. Another possibility is that the cytokine changes occurred secondary to the insertion of the spinal catheter, although other studies in this area consider this to be unlikely^11^. The anaesthetic regimen used in this study was not uniform and the anaesthetic data were incomplete. A useful future study could look at the differences in cytokine responses in those receiving just a spinal anaesthetic versus a spinal and general anaesthetic in patients with a fractured neck of femur. However, previous work comparing PND between patients undergoing cardiac surgery with either GA or local anaesthesia showed no difference between the two groups, suggesting that neuroinflammation is driven by surgery, rather than by anaesthesia^32^.

Cytokine activation is only part of the neuroinflammatory process after surgery^1^. Other studies in this field have looked at the CSF to serum albumin ratio before and after surgery as a marker of BBB integrity, which was not investigated here^33,11,28^. A more detailed analysis of the CSF including cell count levels and the immunoglobulin subtypes IgM, IgG and IgA could be performed in future studies, as these would also have relevance in understanding the role neuroinflammation plays in PND^34^. Furthermore, we did not investigate markers of neuronal injury, such as S-100B, t-tau, neuronal specific enolase (NSE) and neurofilament light chain protein (NFL), which other studies have ﻿investigated^ 35^ This was not possible within the scope of this study, however, future studies will need to examine these biomarkers and their relationship with inflammatory cytokines and the genesis of delirium.

A further limitation is the lack of cognitive function testing used in this study. Patients completed an abbreviated mental test score (AMTS) pre-operatively, but underwent no formal post-operative cognitive function testing. There are some difficulties associated with cognitive function testing in patients with a fractured neck of femur. Pre-operatively there is time pressure to take patients to theatre quickly, with best practice guidelines stating that patients should be operated upon within 36 h^14^. This limits the time in which cognitive assessments can be undertaken. Furthermore, the pain associated with a fractured neck of femur can be severe, meaning that results may be potentially confounded by experiences of pain, or the side effects of morphine-based medication. Despite these difficulties, formal cognitive testing pre- and post-operatively should form part of the protocol for future studies in this area. The 4AT test is a fast, sensitive and specific method of screening for delirium in hospitalised older patients, and would be an appropriate tool to be used^36^.

Finally, the additional wrist fractures sustained in three participants is a confounding factor, as are the variations in long-term medication use and medical co-morbidities.

## Conclusions

A key conclusion from this study is that surgery causes a large increase in cytokines in the CSF which are not detected in the plasma. Fracture alone does not appear to drive cytokine increases, although we did not directly measure pre-fracture levels. There were no correlation between levels of cytokines in the plasma or CSF prior to surgery, but a strong correlation between CSF cytokines on day one post-surgery. There were no correlations between plasma and CSF cytokines, suggesting measuring plasma cytokines cannot give an indication of patients having a greater neuroinflammatory response.

This pilot study offers the theoretical basis for the design of carefully designed clinical studies with plasma and CSF sampling at pre-defined intervals to study the nature of inflammation in relation to surgery, anaesthesia and delirium. Acute studies of this nature could identify biomarkers to assess, prevent and treat PND. These studies should be multicentre and not exclude non-English speakers to ensure greater patient numbers. Biofluid samples should be taken at more time points and with formal cognitive assessments of PND.

## Supplementary Information

Supplementary Tables.

## Data Availability

The dataset supporting the conclusions of this article is available from the corresponding author upon reasonable request.

## Abbreviations

AMTS: Abbreviated mental test score
BBB: Blood-brain barrier
CNS: Central nervous system
CSF: Cerebrospinal fluid
IFN-γ: Interferon gamma
IL: Interleukin
LLD: Lower limit of detection
PND: Peri-operative neurocognitive disorders
T1: Time-point 1 (pre-operation)
T2: Time-point 2 (post-operation, day of surgery)
T3: Time-point 3 (post-operation, day after surgery)
TNF-α: Tumour necrosis factor alpha
ULD: Upper limit of detection
