# Supplementary material for: Cytokine changes in cerebrospinal fluid and plasma after emergency orthopaedic surgery
Source: Sci Rep. 2022 Feb 9;12:2221. doi: 10.1038/s41598-022-06034-9 (PMC8828833; doi:10.1038/s41598-022-06034-9)
Supplement: Supplementary file 1 — Supplementary Tables. [file 41598_2022_6034_MOESM1_ESM.docx]

**Supplementary material for the manuscript ‘Cytokine changes in cerebrospinal fluid and plasma post-emergency orthopaedic surgery’**

**Supplementary Table 1: Operation and anaesthetic type**

| **ID** | **Hrs to Op** | **Op type** | **Type of anaesthesia** | **Intrathecal medication** | **Intravenous medication** |
| --- | --- | --- | --- | --- | --- |
| **PO2** | 26 | HA | Spinal block | Bupivacaine 25mg, Diamorphine 0.25mg |  |
| **PO3** | 39 | HA | Spinal block & sedation | Bupivacaine 15mg, Diamorphine 0.3mg | Ketamine 30mg,  Propofol 20mg |
| **PO9** | 14 | HA | Spinal block & GA | Bupivacaine 7.5mg, Diamorphine 0.4mg | Propofol 160mg, Fentanyl 75mcg, Diamorphine 3mg, Sevoflurane |
| **PO10** | 24 | HA | N/A | N/A | N/A |
| **PO11** | 22 | HA | Spinal block & GA | Diamorphine 0.3mg | Propofol 200mg, Fentanyl 100mcg, Sevoflurane |
| **PO12** | 16 | DHS | Spinal block & sedation | Levobupivacaine 10mg,  Fentanyl 25mcg | Fentanyl 50mcg, Midazolam 2mg |
| **PO13** | 21 | THR | Spinal block & sedation | Bupivacaine 15mg,  Diamorphine 0.5mg | Fentanyl 75mcg, Midazolam 2mg |
| **PO14** | 18 | HA | Spinal block & GA | Bupivacaine 7.5mg, Diamorphine 0.5mg | Fentanyl 50mcg, Propofol 200mg |
| **PO15** | 24 | DHS | Spinal block & sedation | Bupivacaine 12.5mg, Diamorphine 0.3 mg | Ketamine 10mg, Fentanyl 15mcg |
| **PO19** | 27 | HA | N/A | N/A | N/A |
| **PO20** | 94 | CS | N/A | N/A | N/A |

**Key**: Hrs to Op = Hours from admission to operation start time, HA = Hemiarthroplasty, DHS = Dynamic hip screw, THR = Total hip replacement, CS = Cannulated screws, GA = General anaesthetic, N/A = Not available

**Supplementary Table 2: Participants post-operative complications**

| **ID** | **Opiate (mg)** | **Hb drop**  **(g/dL)** | **Creat rise (%)** | **LoS** | **D/C venue** | **Carer hrs at d/c** | **Days of delirium** | **Post op complication** |
| --- | --- | --- | --- | --- | --- | --- | --- | --- |
| **PO2** | 16 | -35 | -22 | 8 | Home | 0 | 0 |  |
| **PO3** | 0 | -22 | -26 | 21 | Rehab | 24 | 0 | UTI |
| **PO9** | 23 | -61 | 10 | 42 | Rehab | 24 | 0 | MI, haematoma |
| **PO10** | 29 | -23 | 8 | 5 | Home | 1 | 0 |  |
| **PO11** | 6 | -36 | -20 | 20 | Home | 4 | 0 |  |
| **PO12** | 10 | -9 | -5 | 8 | Home | 2 | 0 | *CSF headache |
| **PO13** | 6 | -30 | 5 | 9 | Home | 0 | 0 | PE |
| **PO14** | 16 | -4 | -15 | 6 | Home | 0 | 0 |  |
| **PO15** | 23 | -30 | -9 | 21 | Home | 2 | 0 |  |
| **PO19** | 15 | -21 | -4 | 15 | Home | 0 | 0 |  |
| **PO20** | 0 | -21 | -18 | 10 | Rehab | 24 | 2 |  |

**Key**: *CSF headache = a headache secondary to low cerebrospinal (CSF) levels, Hb drop = Drop in haemoglobin following surgery, Creat rise (%) = Percentage rise in creatinine following surgery, LoS = Length of stay in hospital, D/c = Discharge, Rehab = Community rehabilitation facility, Carer hrs at d/c = Number of hours of care provided at discharge from hospital, Days of delirium = Number of days that delirium was present following surgery, UTI = Urinary tract infection, MI = Myocardial infarction, PE = Pulmonary embolism.

**Supplementary Table 3: Intercorrelation between CSF cytokines at T3 (day 1 post-operation)**

| **Intercorrelation between cytokines** | **Adjusted-p** | **R value** |
| --- | --- | --- |
| IL-1β and IL-2 | <0.05 | 0.85 |
| IL-1β and IL-8 | <0.001 | 0.95 |
| IL-1β and TNF-α | <0.01 | 0.91 |
| IL-2 and TNF-α | <0.05 | 0.86 |
| IL-2 and IL-8 | <0.05 | 0.88 |
| IL-10 and IL-2 | <0.01 | 0.93 |
| IL-10 and IL-4 | <0.01 | 0.9 |
| IL-10 and IL-12p70 | <0.05 | 0.86 |
| IL-10 and IL-1β | <0.05 | 0.87 |
| IL-12p70 and IL-4 | <0.001 | 0.97 |
| IL-13 and IL-1β | <0.01 | 0.9 |
| IL-13 and IL-2 | <0.05 | 0.86 |
| IL-13 and IL-8 | <0.01 | 0.9 |
| IL-13 and TNF-α | <0.05 | 0.94 |
| IFN-γ and IL-2 | <0.01 | 0.91 |
| IFN-γ and IL-4 | <0.05 | 0.85 |
| IFN-γ and IL-10 | <0.05 | 0.87 |
| IFN-γ and IL-12p70 | <0.05 | 0.85 |

**Key**: Only statistically significant changes shown (adjusted-*p* <0.05)

**Legend:** A table showing the statistically significant intercorrelation findings between cerebrospinal fluid (CSF) cytokines at T3 (day 1 post-operation).
